# Soil Microbiomes Across Depth and Ecosystems in Dubai, UAE: Potential Environmental Signatures for Forensic Geolocation

**DOI:** 10.1111/1758-2229.70403

**Published:** 2026-08-14

**Authors:** Abdulla Albastaki, Mohammed Naji, Mohamed Moussa, Judith Smith

**Affiliations:** ^1^ General Department of Forensic Science and Criminology Dubai Police HQ Dubai UAE; ^2^ School of Law and Policing University of Lancashire Lancashire UK; ^3^ International Center for Biosaline Agriculture Dubai UAE; ^4^ Department of Integrative Agriculture, College of Agriculture and Veterinary Medicine United Arab Emirates University Al Ain UAE; ^5^ Soil and Water Research Department, Nuclear Research Center Egyptian Atomic Energy Authority Cairo Egypt

**Keywords:** 16S rRNA gene sequencing, arid soils, microbial diversity, soil microbial communities

## Abstract

Soil microbial communities exhibit strong sensitivity to environmental gradients, yet their distribution across depth and land‐use types in hyper‐arid environments remains poorly characterised. Using whole‐genome shotgun metagenomics via Oxford Nanopore Technologies long‐read sequencing, we profiled soil microbial communities across six contrasting land‐use sites in Dubai, UAE: urban, industrial (two locations), marine, desert and agricultural, where each sampled at three depth intervals (0–25 cm, 25–50 cm and 50–100 cm). Marine soils exhibited extreme salinity (EC 23.7–30.7 dS m^−1^) and the highest organic matter content (1.19%–1.76%), while desert soils were nutrient‐poor with minimal salinity. Actinomycetota and Pseudomonadota co‐dominated across all sites, collectively accounting for 77%–96% of classified sequences. Actinomycetota prevailed in undisturbed desert horizons (up to 53.4%), while Pseudomonadota dominated nutrient‐enriched environments, reaching 69.4% at industrial sites. A notable compositional reversal was observed in the desert deep horizon (50–100 cm), where Pseudomonadota increased to 56.8%, departing from the expected oligotrophic depth gradient. PERMANOVA confirmed land use as the primary driver of community composition (*p* = 0.001), with depth exerting a secondary but significant effect (*p* ≤ 0.01). NMDS ordination revealed strong site‐specific clustering, with each environment harbouring a distinctive microbial fingerprint with promising forensic geolocation potential.

## Graphical abstract

Soil microbiomes from diverse Dubai environments were profiled using a high‐throughput sequencing approach. Distinct microbial signatures were identified across sites, enabling comparative taxonomic and diversity analyses. These microbial fingerprints demonstrate potential for forensic geolocation, supporting the use of soil microbiome profiling as a tool in forensic investigations.

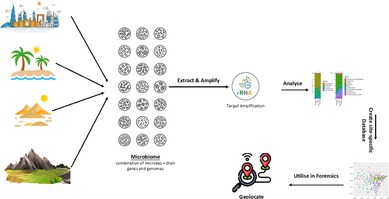

## 1 Introduction

Soil microbial communities are fundamental to ecosystem functioning in arid lands, driving processes such as organic matter turnover and nutrient cycling even under extreme conditions (Maestre et al. 2015). Both soil depth and hyper‐aridity are known to shape these communities. Recent synthesis shows that microbial composition and functional potential can vary substantially with depth, as deeper soils often harbour distinct taxa adapted to low oxygen and carbon availability (Naylor et al. 2022). Yet, traditional studies have overwhelmingly focused on surface soils, overlooking the biological richness of deeper layers (Naylor et al. 2022). In agricultural settings, for instance, microbial richness and diversity tend to decrease with increasing soil depth (Hao et al. 2021), accompanied by shifts in dominant taxa (e.g., Proteobacteria becoming more dominant in subsoils) (Hao et al. 2021).

Hyper‐arid deserts impose additional constraints—minimal moisture and intense heat at the surface limit life to highly specialised extremophiles (Makhalanyane et al. 2015). Notably, even in one of the world's driest regions (Atacama Desert), microbial communities were found to persist at depth, structured by gradients in moisture and salinity (Fuentes et al. 2022; Pérez‐Valera et al. 2019). Depth, in conjunction with edaphic factors like soil moisture, soil salinity and nutrients, plays a critical role in driving community composition in hyper‐arid soils (Fuentes et al. 2022).

These findings underscore the importance of considering soil depth in microbial surveys of arid ecosystems. Land use and anthropogenic disturbance further modulate soil microbiota. Conversion of natural deserts to agriculture introduces water and organic inputs that can dramatically alter community structure. For example, long‐term organic farming on Egyptian desert soil increased overall microbial diversity and enriched plant‐beneficial taxa (e.g., *Bacillus* spp.) but led to the loss of many native extremophiles (Köberl et al. 2013; Berg et al. 2017).

A study mentioned that 30 years of cultivation raised the Shannon diversity from 7.9 in native desert to 11.2 in farmed soil, while desert‐adapted genera (e.g., *Deinococcus*) disappeared (Köberl et al. 2013; Berg et al. 2017). Beyond agriculture, other land uses such as urban and industrial development sites impose chemical and physical disturbances that reshape microbial communities. Recent work in semi‐arid region of China showed that soil bacterial diversity and network complexity differ markedly among land‐use types, with intensive uses (e.g., tilled farmland) generally reducing complexity relative to less disturbed shrublands (Teresa Mabiala et al. 2020).

Wu et al. (2024) similarly reported that anthropogenic factors can drive predictable shifts in community composition, often selecting for microbes tolerant of fertilisers, pollutants, or other human inputs (Wu et al. 2024). Taken together, these studies highlight that both natural gradients (depth, moisture) and anthropogenic land use strongly influence soil microbiota, making it crucial to study their combined effects in understudied environments like the hyper‐arid soils of the Arabian Peninsula.

Advances in DNA sequencing now enable comprehensive profiling of these complex microbial communities. In particular, whole‐genome shotgun metagenomics provides an unbiased assessment of all DNA in a sample, capturing bacteria, archaea, fungi and viruses as well as functional genes. This approach surpasses targeted 16S rRNA surveys by revealing not only taxonomic diversity but also the genetic potential (e.g., metabolism, stress resistance) present in the microbiome.

Here we employ Oxford Nanopore Technologies (ONT) long‐read sequencing to interrogate Dubai's soil microbiome across varying depths and land uses. ONT's real‐time, portable sequencing is well‐suited for studying extreme environments, as it can generate long reads that improve genome assembly from novel or low‐abundance organisms (Lu et al. 2025). Long‐read metagenomics has been shown to overcome many limitations of short‐read methods, resolving repetitive genomic regions and enabling retrieval of near‐complete microbial genomes from environmental samples (Lu et al. 2025).

Moreover, ONT sequencing is relatively low‐cost and rapid, with the GridION platform yielding hundreds of gigabases of data per run in real time (Lu et al. 2025; Zaheer et al. 2018). Ultimately, the rich metagenomic data allow us to link community structure with ecosystem function under different land uses.

While this study primarily focused on examining how soil depth and land use influence microbial composition in a hyper‐arid desert environment, it did not explore functional genes. Instead, the work aimed to assess the potential of microbial community profiles for environmental differentiation, including whether depth‐related variation could be observed. Soil microbial fingerprints are increasingly recognised as a tool for forensic geolocation, since each environment selects for a unique consortium of microbes (Tripathi et al. 2024). In principle, the microbial DNA profile of a soil sample could help identify its provenance, distinguishing an agricultural field from a natural desert or even pinpointing a specific locale (Tripathi et al. 2024).

## 2 Materials and Methods

### 2.1 Study Sites and Soil Sampling

**FIGURE 1:**
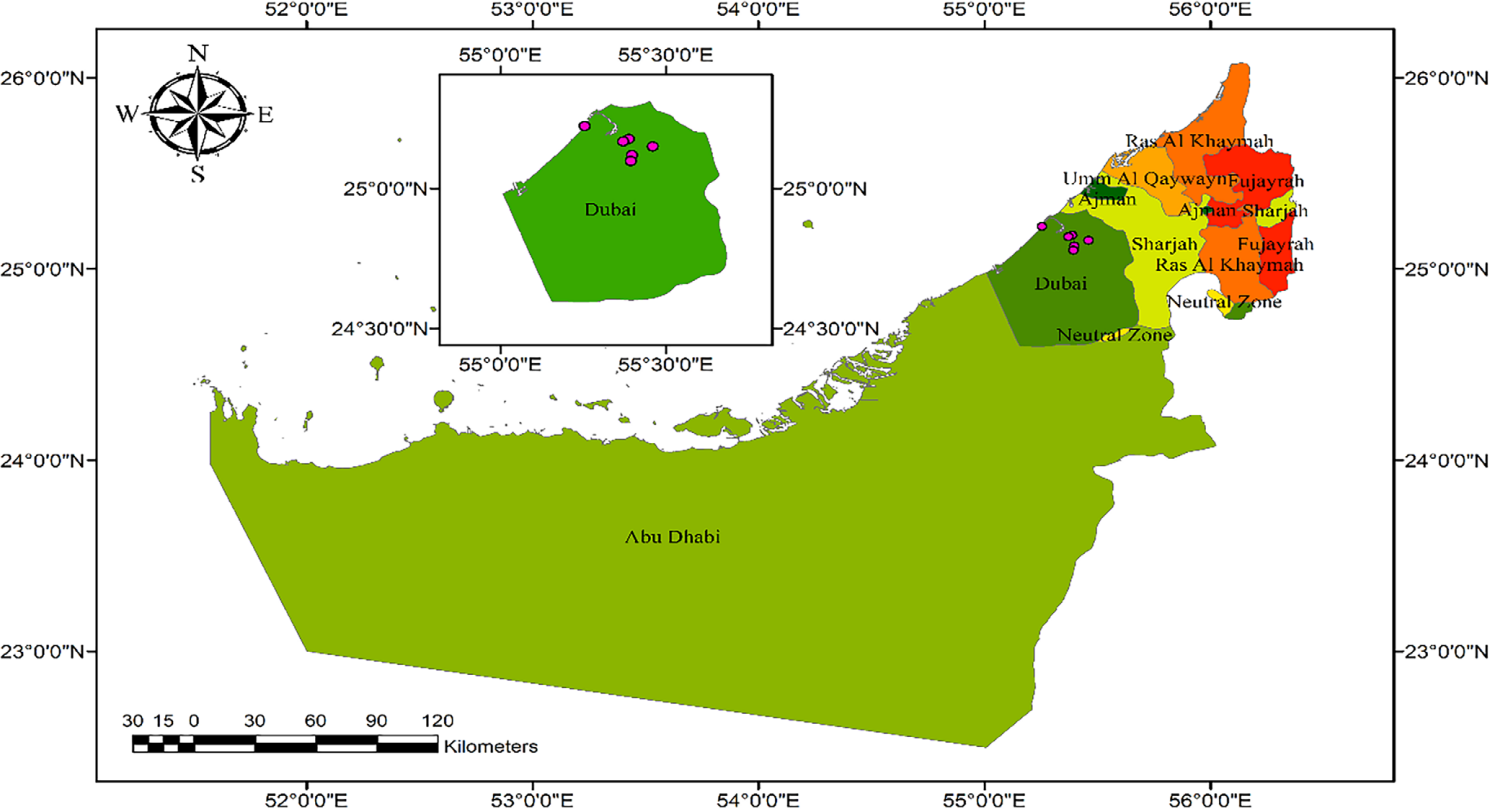
Spatial distribution of urban, industrial, marine, agricultural and desert soil samples collected across Dubai, UAE.

This study was conducted across six distinct land‐use sites in Dubai, United Arab Emirates, selected to represent a range of environmental and anthropogenic conditions. The selected sites included: (1) an active agricultural field; (2) a natural desert environment; (3) a coastal (marine‐influenced) site; (4) two industrial sites (Industrial A: industrial facilities; Industrial B: car garages and industrial warehouses); and (5) an urban soil as shown in Figure 1. At each site, soil samples were collected at three depth intervals (0–15) cm, (15–50) cm and (50–100) cm to capture vertical stratification. Three replicate samples were taken per depth (using adjacent points ~5 to 10 m apart). Soil cores were extracted using sterile stainless‐steel augers. The central portion of each depth segment was carefully isolated and transferred into sterile polyethylene bags. To prevent cross‐contamination, all sampling tools were thoroughly cleaned and sterilised between each sample collection. The International Center for Biosaline Agriculture (ICBA) field team performed the sampling in accordance with standard biosecurity protocols. Immediately after collection, samples were kept on ice packs, transported to the laboratory and stored at −20°C until DNA extraction.

### 2.2 Soil Analysis

Soil pH was determined in a 1:2.5 soil‐to‐distilled water suspension by mixing 10 g of air‐dried, sieved (< 2 mm) soil with 25 mL of distilled water, shaking intermittently for 30 min and measuring with a calibrated pH metre according to Jackson 1973 (Jackson 1973). Electrical conductivity (EC) was measured in the same suspension using a conductivity metre (Mettler‐Toledo, AG, 8603 Schwerzenbach, Switzerland) and expressed as dS m^−1^ (Rhoades 1996). Whilst soil organic matter (OM) content was analysed by the Walkley–Black wet oxidation method, in which soil organic carbon was oxidised with potassium dichromate and concentrated sulphuric acid, and the remaining dichromate was titrated with ferrous ammonium sulphate, OM was calculated from the oxidisable organic carbon content (Walkley and Black 1934).

### 2.3 DNA Extraction and Quality Control

Microbial DNA was extracted from each soil sample using the DNeasy PowerSoil Pro Kit (Qiagen, Cat. No. 47014) following the manufacturer's protocol optimised for high‐efficiency lysis of soil microbes. Approximately 0.25 g of each soil (well‐mixed) was used per extraction to ensure representativeness. The kit's mechanical and chemical lysis steps were applied to break up soil aggregates and release DNA from bacterial and fungal cells. After extraction, DNA yields and purity were assessed. DNA concentration was measured with a Qubit 4 fluorometre using the high‐sensitivity dsDNA assay (Qubit dsDNA HS Kit, Thermo Fisher Scientific, Cat. No. Q32851). All samples yielded sufficient DNA for sequencing. In addition, the DNA fragment size distribution was evaluated on an Agilent 4200 TapeStation system using the Genomic DNA ScreenTape assay (Agilent Technologies, Cat. No. 5067–5365). DNA extracts were stored at −20°C until library preparation.

### 2.4 Metagenomic Library Preparation and Nanopore Sequencing

Whole‐genome shotgun libraries were prepared for each sample using the Oxford Nanopore Technologies Rapid Barcoding Sequencing Kit (SQK‐RBK004). This kit allows rapid tagmentation of DNA and addition of barcoded adapters, enabling multiplexing of samples. The prepared libraries were loaded onto R10.4.1 flow cells and sequenced on an ONT GridION platform. Real‐time basecalling was carried out using Dorado basecaller (high‐accuracy model) (Oxford Nanopore Technologies PLC, 2026; version 1.3.1) (Oxford Nanopore Technologies PLC 2026a) integrated in the MinKNOW software (Oxford Nanopore Technologies PLC, 2026; v25.05.14) (Oxford Nanopore Technologies PLC 2026b). Sequencing run was performed for ~48 h. Across all samples, this amounted to several hundred gigabases of sequence data, providing deep coverage of the soil metagenomes.

### 2.5 Taxonomic and Functional Profiling

High‐quality reads were then analysed using Oxford Nanopore's EPI2ME platform for metagenomic classification. In particular, we employed the ‘Metagenomics’ workflow, which performs real‐time taxonomic identification of each read. This workflow utilises the Kraken2 classification algorithm (Wood et al. 2019) with ‘core_nt’ database as the reference for assignment. The output for each sample was a taxonomic profile of bacteria, archaea and eukaryotic microbes present, including relative abundance estimates. To assess community composition, we compiled read counts per taxon and computed alpha‐diversity metrics (richness, Shannon–Wiener index) for each sample.

### 2.6 Statistical Analysis

All statistical analyses were conducted in RStudio (R version 4.2.2) with appropriate packages for community ecology (R Core Team 2024). Alpha‐diversity indices (observed species, Shannon diversity) were calculated using the vegan package (Oksanen and Simpson 2023), and differences across depths and sites were tested by analysis of variance (ANOVA). Beta‐diversity was examined through Bray–Curtis dissimilarity and visualised via non‐metric multidimensional scaling (NMDS) ordinations. We employed PERMANOVA (Permutational Multivariate ANOVA, using the adonis function in vegan) to quantitatively assess the influence of depth, land use and their interaction on community composition dissimilarities. Graphical outputs (boxplots of diversity, NMDS plots, etc.) were generated with ggplot2 (Wickham 2016). These statistical approaches enabled us to rigorously test our central hypotheses regarding microbial diversity patterns across the depth profile and between contrasting land uses in a hyper‐arid environment. All results were organised and plotted for interpretation in the subsequent sections of the manuscript.

## 3 Results

Soil physicochemical analysis revealed distinct gradients across land‐use types and depths (Table 1). Marine soils exhibited extreme salinity (EC 23.7–30.7 dS m^−1^) and the highest organic matter content (OM 1.19%–1.76%), consistent with coastal influence. In contrast, hyper‐arid desert soils showed minimal salinity (EC 0.42–0.60 dS m^−1^) and low OM (0.41%–0.80%), reflecting limited biotic inputs. Agricultural and urban topsoils (0–25 cm) displayed elevated OM (0.81% and 0.70%, respectively), indicative of surface enrichment from anthropogenic activity, with values declining significantly at deeper layers. Industrial sites exhibited irregular EC patterns (1.74–3.60 dS m^−1^), suggesting localised contamination. All soils were alkaline (pH 7.42–8.10), with marine soils reaching peak alkalinity (pH 8.10 at 25–50 cm), aligning with typical carbonate buffering in arid regions. These physicochemical gradients create distinct microhabitats that likely shape microbial community structure and function.

**TABLE 1:** Soil physicochemical properties by depth and land‐use type in Dubai's arid ecosystem.

Soil type	Soil depth	pH	Ec (dS m^−1^)	OM (%)
Urban soil	0–25 cm	7.42	8.06	0.6971
	25–50 cm	7.75	4.32	0.4426
	50–100 cm	7.80	3.04	0.3861
Industrial soil (A)	0–25 cm	7.60	2.141	0.5195
	25–50 cm	7.72	1.769	0.3776
	50–100 cm	7.63	3.6	0.3948
Industrial soil (B)	0–25 cm	7.78	3.07	0.6801
	25–50 cm	7.82	3.40	0.4900
	50–100 cm	7.79	1.740	0.3641
Marine soil	0–25 cm	7.86	23.7	1.1890
	25–50 cm	8.10	24.4	1.6150
	50–100 cm	8.04	30.7	1.7618
Desert soil	0–25 cm	7.80	0.506	0.4441
	25–50 cm	7.74	0.601	0.4067
	50–100 cm	8.03	0.422	0.8025
Agriculture soil	0–25 cm	7.68	2.59	0.8051
	25–50 cm	7.78	0.688	0.5171
	50–100 cm	7.81	0.691	0.4725

*Note:* Where EC refers to the soil electrical conductivity, and OM refers to the soil organic matter.

Taxonomic profiling of the soil metagenomes revealed a consistent co‐dominance of Actinomycetota (formerly Actinobacteria) and Pseudomonadota (formerly Proteobacteria) across all six sites. Together, these two lineages accounted for approximately 77%–96% of classified sequences per sample (combined range across all site–depth combinations), with their relative proportions varying markedly as a function of both site and depth. At the Desert site, Actinomycetota was the dominant phylum in surface and mid‐depth horizons, comprising approximately 50.6% at 0–25 cm and 53.4% at 25–50 cm, while Pseudomonadota contributed 36.8% and 40.1% at those same layers, respectively. This pattern is consistent with the well‐documented enrichment of drought‐tolerant Actinobacteria in undisturbed arid soils globally (Xu et al. 2021; Vásquez‐Dean et al. 2020). However, a notable reversal occurred in the deepest desert horizon (50–100 cm), where Pseudomonadota rose to 56.8% while Actinomycetota declined to 38.2%, indicating that the vertical dominance gradient in the desert does not follow a simple linear trend and warrants further investigation. The Marine site was characterised by pronounced Pseudomonadota dominance across all depths (58.7% at 0–25 cm, 68.1% at 25–50 cm and 64.9% at 50–100 cm), with Actinomycetota correspondingly reduced (28.3%, 23.8% and 31.1%, respectively), reflecting the selective advantage conferred on halotolerant Proteobacterial taxa under conditions of elevated salinity. The agricultural surface horizon likewise exhibited a Pseudomonadota‐rich profile (56.2% at 0–25 cm), consistent with reports that irrigation and fertilisation in arid‐zone cultivated soils favour copiotrophic, nutrient‐responsive taxa (Xu et al. 2021), though Pseudomonadota declined progressively with depth (44.5% at 25–50 cm; 42.0% at 50–100 cm) as Actinomycetota increased correspondingly (34.9% → 43.7% → 46.4%).

**FIGURE 2:**
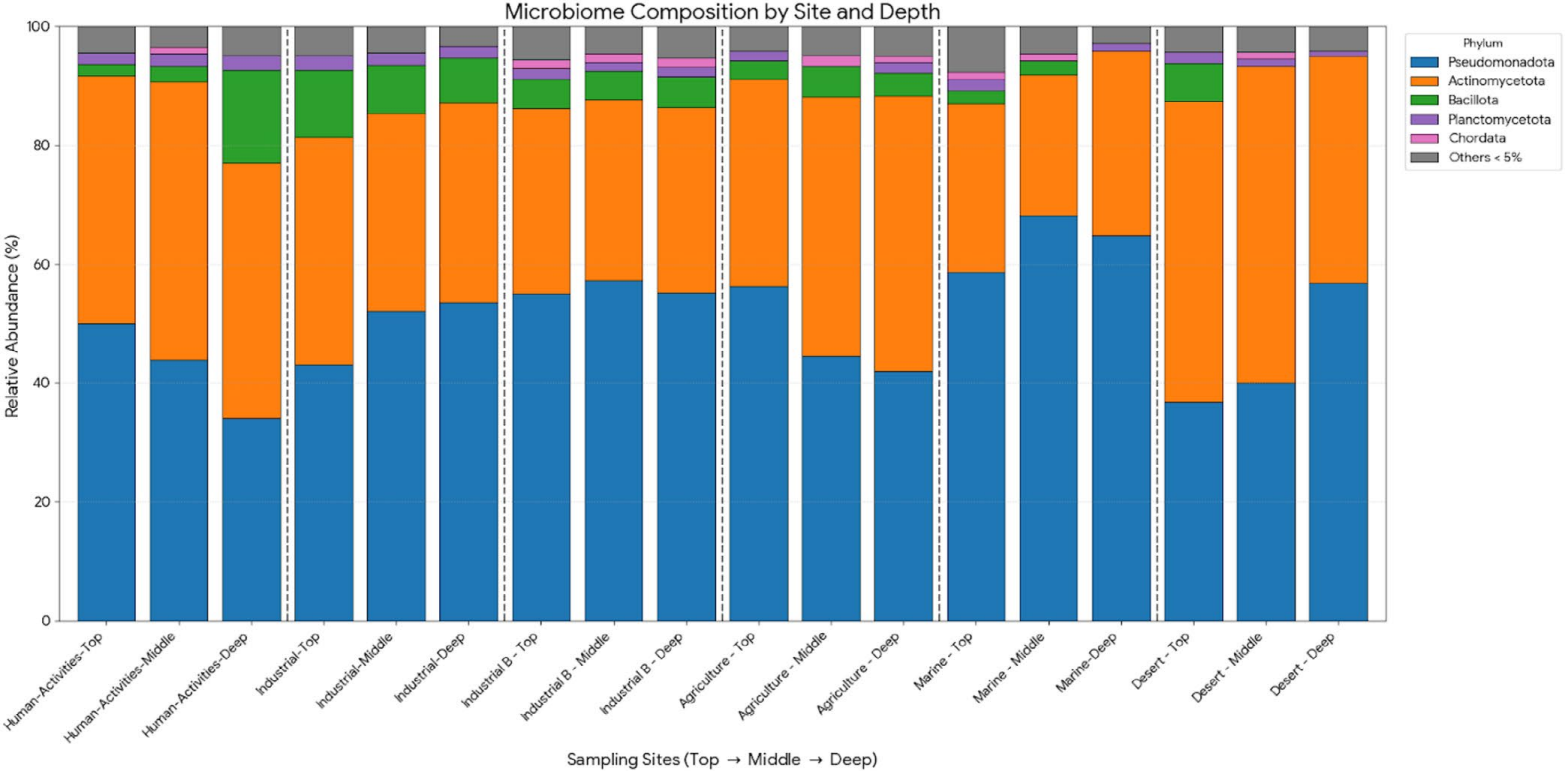
Phylum‐level composition of soil microbial communities across the six Dubai sites at three depth intervals (top: 0–25 cm, middle: 25–50 cm, deep: 50–100 cm). Each bar represents the relative abundance of major bacterial phyla in a given site and depth. Actinomycetota (Actinobacteria) and Pseudomonadota (Proteobacteria) together account for the majority of sequences (≈70%–90%). Minor phyla (e.g., Bacillota, Planctomycetota, Acidobacteriota) occur at low frequencies.

**FIGURE 3:**
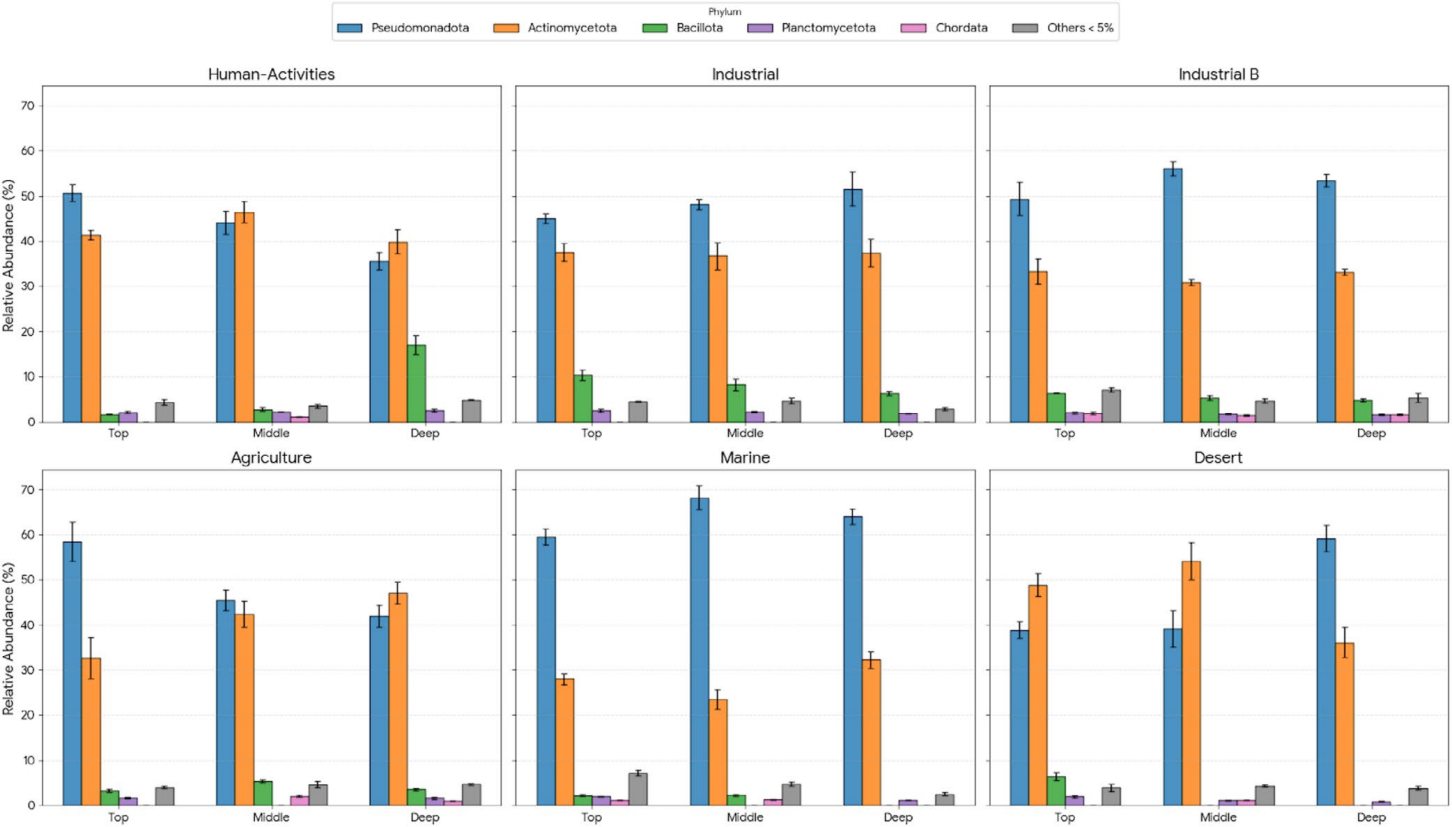
Phylum‐level microbial composition across sampling sites and depths. Each panel represents a specific environment, with bars showing the mean relative abundance across three sampling depths (top: 0–25 cm, middle: 25–50 cm, deep: 50–100 cm). Error bars indicate the 95% Confidence Interval (*n* = 3) derived from biological triplicates for each sample. Less abundant phyla are consolidated into the ‘Others < 5%’ category. The data demonstrate site‐specific vertical stratification and variability in microbial dominance.

Beyond the two dominant phyla, several minor lineages contributed at low but consistent abundances. Bacillota (Firmicutes) represented a minor fraction across most samples (generally < 7%), with one notable exception: the Human Activities deep horizon (50–100 cm) showed a pronounced enrichment of Bacillota at 15.6%, substantially exceeding values at the surface (1.9%) and mid‐depth (2.8%) layers of the same site. This depth‐associated spike may reflect the selective enrichment of endospore‐forming taxa under conditions of increasing resource limitation and physical disturbance at depth. Industrial sites also showed slightly elevated Bacillota at surface horizons (Industrial A top: 6.2%; Industrial B top: 4.4%), consistent with the capacity of spore‐formers to persist under chemically disturbed conditions. Planctomycetota, Acidobacteriota, Gemmatimonadota and Nitrospirota were each present only as trace constituents across sites. Notably, sequences attributable to Euryarchaeota were detected in select surface samples, including the Desert (0–25 cm, ~1.1%) and Marine (0–25 cm, ~4.8%) surface horizons, indicating the localised occurrence of halophilic and xerophilic archaea under the most extreme near‐surface conditions. Their absence from deeper layers suggests that extreme physicochemical conditions at the soil surface selectively sustain a small but ecologically relevant archaeal component. Phylum‐level patterns are summarised in Figure 2, while site‐ and depth‐resolved profiles with 95% confidence intervals are presented in Figure 3.

At the family level, no single taxon dominated uniformly across the dataset; rather, community composition was taxonomically heterogeneous, with several families appearing consistently at low to moderate relative abundances. Among the more frequently detected families were *Comamonadaceae* (Betaproteobacteria, noted for metabolic versatility), *Enterobacteriaceae* (Gammaproteobacteria, encompassing both soil and enteric‐associated members), *Marinobacteraceae* (Gammaproteobacteria, adapted to saline conditions), *Paenibacillaceae* (Firmicutes, spore‐forming taxa) and *Streptomycetaceae* (Actinobacteria, filamentous decomposers). *Marinobacteraceae* sequences were effectively absent from inland sites yet were comparatively enriched at the Marine 25–50 cm horizon (Marinobacter‐related reads ~0.7%), consistent with the saline character of that site. *Streptomycetaceae* were detected across all environments but showed slightly elevated proportions in deeper or drier samples (e.g., ~2.6% in the Agriculture 50–100 cm horizon), in accordance with their role as drought‐resistant decomposers. *Enterobacteriaceae* remained below 1% in most samples but reached approximately 3.5% in the Desert 50–100 cm horizon, coinciding with the broader Pseudomonadota spike observed at that depth and possibly reflecting an opportunistic enrichment associated with a localised pulse of organic substrate, though this interpretation warrants further investigation.

Statistical analyses confirmed that both land use and soil depth exerted significant and independent influences on microbial community composition. PERMANOVA analysis of Bray–Curtis dissimilarities indicated that site identity (as a proxy for land use and inherent environmental conditions) was the primary determinant of community variation (*p* = 0.001), while depth contributed a secondary but statistically significant effect (*p* ≤ 0.01). Two‐way ANOVA applied to the relative abundances of dominant phyla revealed broadly significant depth‐dependent shifts, though the direction of these shifts was not uniform across all sites. In the Agriculture and Human Activities environments, Actinomycetota increased and Pseudomonadota declined progressively from the surface to the 50–100 cm horizon, consistent with the expected enrichment of oligotrophic taxa at depth as carbon and nutrient availability wanes. In the Desert, however, this trend held only in the upper two depth intervals, with a reversal observed at 50–100 cm, where Pseudomonadota increased sharply (56.8%) and Actinomycetota declined (38.2%), suggesting site‐specific factors modulate the depth response in the most arid environment. No significant site × depth interaction was detected overall, indicating that depth‐related compositional gradients were broadly consistent across the majority of contrasting land‐use environments, with the Desert deep horizon representing a notable exception.

**FIGURE 4:**
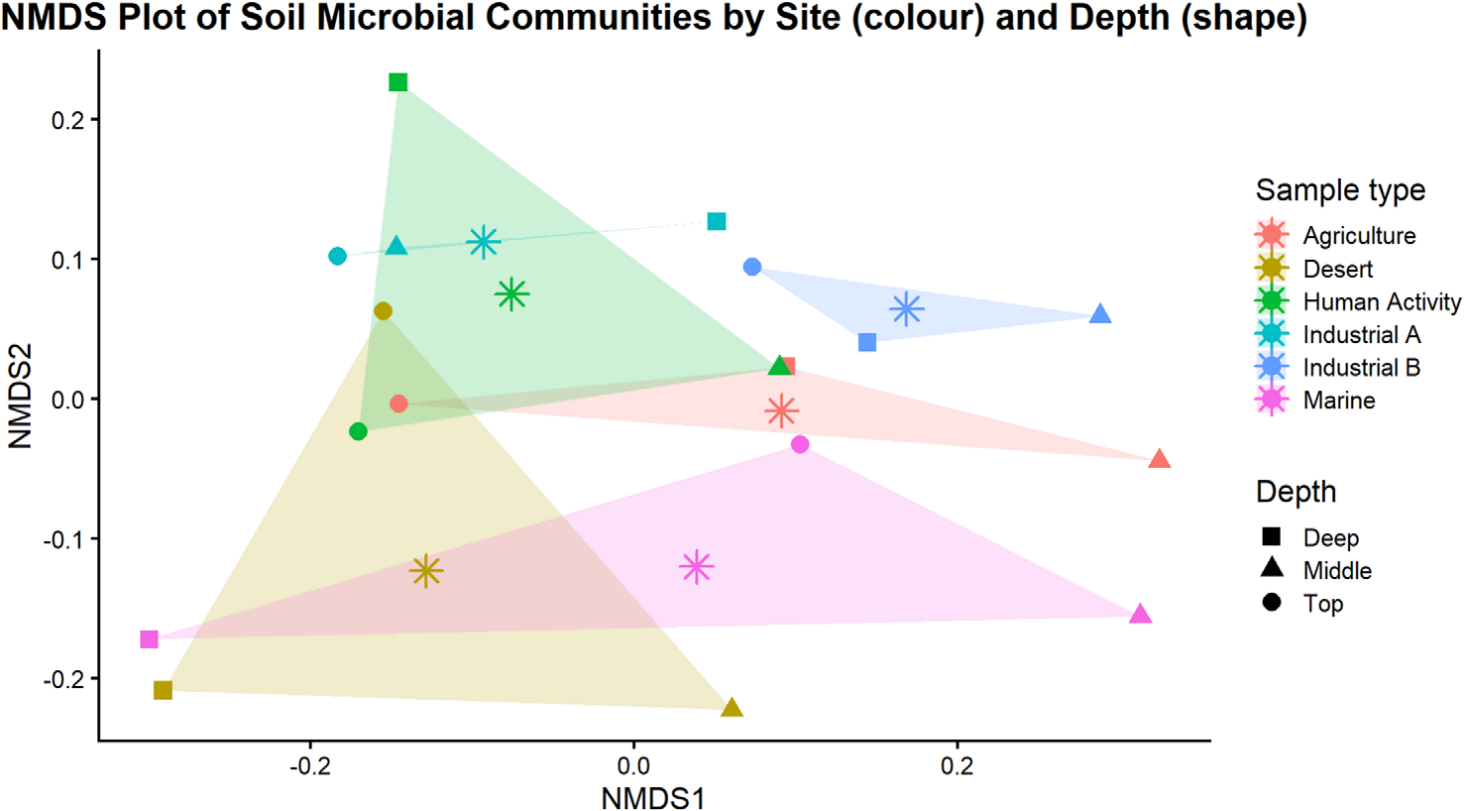
NMDS ordination of soil microbial communities across all samples. Points are coloured by site and shaped by depth (● = 0–25 cm, ▲ = 25–50 cm, ■ = 50–100 cm). Site identity drives the primary clustering, while depth adds a secondary layer of stratification.

**FIGURE 5:**
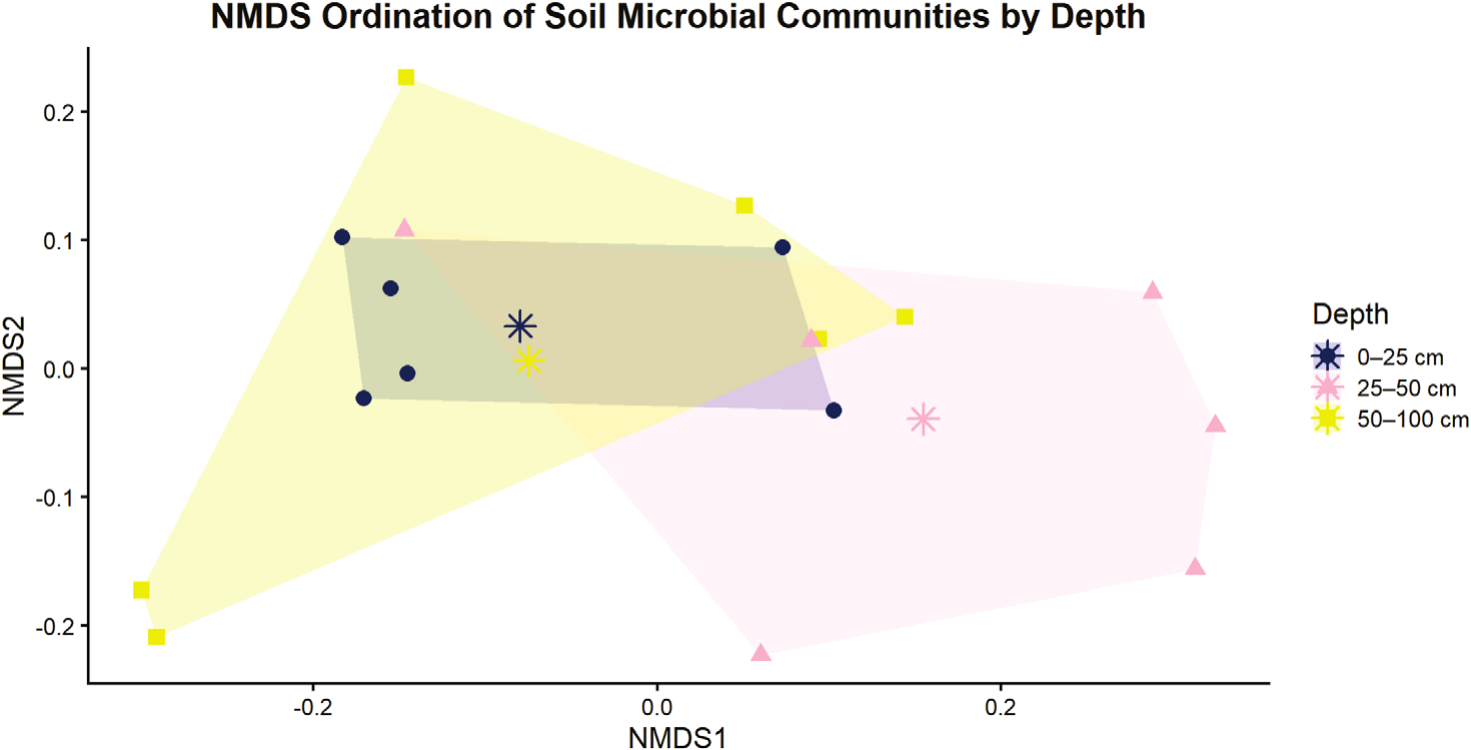
Non‐metric multidimensional scaling (NMDS) plot displaying soil microbial community composition based on Bray–Curtis dissimilarity, coloured by depth (0–25 cm = navy blue, 25–50 cm = pink, 50–100 cm = yellow). Samples cluster according to their vertical stratification, showing compositional differences between surface and deeper soil layers across all sites.

Non‐metric multidimensional scaling (NMDS) ordination of Bray–Curtis dissimilarities provided a visually interpretable summary of the multivariate community differences (Figure 4). The ordination converged to a low stress value, indicating an adequate two‐dimensional representation of the high‐dimensional dissimilarity matrix. Samples clustered most strongly by site identity: replicates from the same location grouped tightly in ordination space regardless of the depth from which they were drawn, while samples from different sites were well separated. Industrial soil replicates formed notably cohesive clusters, consistent with the high and relatively uniform Pseudomonadota dominance observed at those sites across depths (Industrial B: 69.4% at top, 58.8% at mid, 55.2% at deep). Within individual sites—most clearly at the Agriculture location—surface samples (0–25 cm) were offset from deep samples (50–100 cm) along one ordination axis, reflecting the progressive Actinomycetota enrichment with depth at that site. The Desert site exhibited a more complex within‐site pattern, with the deep sample displaced distinctly from the surface and mid‐depth replicates, corresponding to the Pseudomonadota reversal documented at 50–100 cm. Critically, no cross‐site clustering by depth was apparent: there was no emergent grouping of all surface soils irrespective of location, nor of all deep soils, confirming that the site‐specific environmental signal dominated the depth‐related signal at the scale of this study. This result is consistent with forensic soil surveys demonstrating that microbial communities from the same geographic location resemble one another more closely than communities from the same depth at a different site (Gouello et al. 2021). Figure 5 presents the same ordination coloured by depth class, illustrating the secondary depth‐related stratification within the broader site‐driven structure.

## 4 Discussion

This study demonstrates that hyper‐arid soil environments and land‐use practices jointly and significantly shape the soil microbiome in Dubai, with distinct implications for both ecosystem ecology and forensic geolocation. Sequencing was performed using whole‐genome shotgun metagenomics via Oxford Nanopore Technologies long‐read sequencing, which in principle provides access to the full genomic content of each sample, including functional gene repertoires. However, the present study was intentionally scoped to address taxonomic composition across depth and land‐use gradients; functional gene analysis was beyond the scope of this work and is identified as a priority for future investigation. The taxonomic data alone reveal ecologically meaningful patterns that are discussed below. Ecologically, the co‐dominance of Actinomycetota and Pseudomonadota across all sites, accounting for 77%–96% of classified sequences, underscores the resilience and adaptability of these two lineages in extreme environments. Actinomycetota were particularly prominent in the Desert site, comprising approximately 50.6% and 53.4% of sequences at the surface and mid‐depth horizons, respectively, consistent with the well‐documented drought tolerance of this phylum. Prior studies of hyper‐arid deserts have reported Actinobacteria comprising as much as 70%–80% of the microbial community (Neilson et al. 2017), underscoring that they are frequently the keystone survivors in environments too harsh for many other bacteria. Although Actinomycetota proportions in our Dubai soils did not reach those extreme levels, their prevalence in the drier and deeper layers reinforces their ecological primacy under resource‐limited conditions. Their capacity for sporulation, filamentous growth and production of UV‐protective pigments equips them to persist through prolonged desiccation, contributing to basal nutrient cycling even during rainless periods.

The substantial representation of Pseudomonadota (Proteobacteria) in nutrient‐enriched environments, particularly the surface horizons of the Agriculture (56.2%), Human Activities (50.0%), Marine (58.7%) and both Industrial sites (Industrial A: 58.8%; Industrial B: 69.4%) highlights the responsiveness of copiotrophic taxa to resource availability. The Agriculture site, subjected to irrigation and fertilisation, showed a Proteobacteria‐rich topsoil, consistent with reports from other arid‐zone studies where conversion of native dryland to cultivation increases the relative abundance of Proteobacteria and other nutrient‐responsive groups (Xu et al. 2021). At the Industrial B site, Pseudomonadota dominance was particularly pronounced across all depth intervals (69.4% at top, 58.8% at mid, 55.2% at deep), possibly reflecting the persistent availability of hydrocarbon‐derived carbon substrates or other anthropogenic inputs that selectively sustain copiotrophic Proteobacterial lineages, including families such as *Burkholderiaceae* and *Comamonadaceae* known to tolerate chemical contamination. The Human Activities site showed a community profile intermediate between the Desert and Agriculture extremes—Actinomycetota remained abundant (41.7% at surface) while Pseudomonadota also contributed substantially (50.0%) suggesting that moderate anthropogenic influence is sufficient to shift community composition away from a purely drought‐specialist assemblage without fully replacing it. These site‐specific patterns are reinforced by the underlying physicochemical data: the Desert site had the lowest organic matter and electrical conductivity values, consistent with conditions favouring drought‐tolerant taxa, while the Agricultural and Urban soils exhibited higher OM content and slightly elevated EC, reflecting organic inputs and irrigation that favour copiotrophic proliferation (Lauber et al. 2009).

The Marine site stands apart from all other environments sampled, exhibiting a consistently Pseudomonadota‐dominated profile across all three depth intervals (58.7%, 68.1% and 64.9% at surface, mid and deep horizons, respectively) and the lowest Actinomycetota proportions recorded at any site at mid‐depth (23.8%). The elevated EC values at this site (23.7–30.7 dS m^−1^) create a strongly saline microhabitat that selects for halotolerant Proteobacterial lineages, most notably *Marinobacteraceae*, which were effectively absent from all inland sites but comparatively enriched at the Marine 25–50 cm horizon. Furthermore, sequences attributable to Euryarchaeota were detected at the Marine surface horizon (~4.8%), indicating the presence of halophilic archaea under the most extreme surface salinity conditions. Taken together, the Marine site illustrates how a single dominant abiotic driver, that is salinity, can override the broader aridity gradient and impose a fundamentally different community structure, one that is highly distinctive and potentially valuable as a forensic environmental signature.

A particularly noteworthy finding is the compositional reversal observed in the Desert deep horizon (50–100 cm), where Pseudomonadota increased markedly to 56.8% while Actinomycetota declined to 38.2%, inverting the dominance pattern observed at the surface (Actino 50.6%, Pseudo 36.8%) and mid‐depth (Actino 53.4%, Pseudo 40.1%) layers. This reversal is inconsistent with the canonical expectation that oligotrophic Actinobacteria progressively accumulate at depth as resources diminish, a pattern observed in the Agriculture and Human Activities sites in this study and reported elsewhere (Rchiad et al. 2022). One plausible explanation is the anomalous enrichment of *Enterobacteriaceae* (~3.5%) detected specifically in the Desert 50–100 cm horizon, coinciding with the Pseudomonadota spike. This may reflect a localised pulse of organic substrate at depth, possibly derived from ancient buried organic matter, a former root channel, or subsurface drainage that temporarily or historically supported fast‐growing, copiotrophic Proteobacterial taxa. Alternatively, the low absolute microbial biomass anticipated in hyper‐arid deep soils means that stochastic colonisation events or minor environmental heterogeneities may exert disproportionate influence on relative abundance profiles. Regardless of mechanism, this reversal highlights that depth‐related compositional gradients in hyper‐arid environments are not universally linear and that site‐specific subsurface conditions can produce locally distinct community signatures that deviate from broader trends.

The pronounced Bacillota enrichment observed in the Human Activities deep horizon (15.6% at 50–100 cm, compared to 1.9% and 2.8% at the surface and mid‐depth layers of the same site) represents a second ecologically significant depth‐dependent anomaly. Bacillota, particularly spore‐forming genera such as *Bacillus* and *Paenibacillus*, are well established as persistence specialists capable of remaining viable in soil for extended periods under unfavourable conditions (Naylor et al. 2023). The accumulation of these taxa at depth at an anthropogenically influenced urban site may reflect the long‐term deposition and survival of spore‐forming bacteria introduced through surface activity, as in foot traffic or urban runoff that have gradually been translocated downward and persist in the resource‐poor subsoil. Industrial surface horizons also showed elevated Bacillota (Industrial A top: 6.2%; Industrial B top: 4.4%), consistent with the capacity of endospore‐formers to withstand chemical disturbance. The depth‐specific Bacillota enrichment at the Human Activities site may therefore serve as a diagnostic feature of long‐term anthropogenic influence that is detectable even below the active surface layer, with potential utility for subsurface contamination assessment.

Across the majority of sites, vertical stratification of microbial communities broadly mirrored the physicochemical gradients documented in the soil profiles. Organic matter and EC were highest at the surface (0–25 cm), driven by plant inputs, irrigation and anthropogenic activity and declined with depth, correlating with the shift towards oligotrophic, stress‐tolerant taxa such as Actinomycetota in deeper layers (Fierer et al. 2003). Such stratification is typical in arid regions, where moisture and nutrient availability decrease markedly with depth, influencing both microbial composition and putative function (Jiao et al. 2019). The absence of significant bioturbation in these hyper‐arid soils where deep‐rooted plants are scarce outside irrigated areas and rainfall infiltration to 1 m is infrequent means that each depth layer can develop and maintain a relatively independent microbial assemblage over time. This physical isolation amplifies the ecological divergence between surface and subsurface communities, producing the compositional gradients captured by the NMDS ordination. The mid‐depth layer (25–50 cm) frequently occupied an intermediate position in ordination space, consistent with a continuous gradient rather than a sharp ecotone between surface and deep communities. The detection of viable communities even at 50–100 cm attests to the ability of desert microbes to persist in place with minimal external input, a phenomenon reported in other hyper‐arid deserts where microbial life has been documented several metres below the surface (Neilson et al. 2017).

From a forensic geolocation perspective, the clear differentiation of microbial communities by site and to a lesser extent by depth, is highly significant. Each site's microbial fingerprint was sufficiently distinctive that PERMANOVA and NMDS ordination could reliably resolve samples by location of origin, even across the relatively confined geographic area of Dubai. In forensic science, it is well established that microbial communities vary with geographic location due to differences in climate, soil chemistry, vegetation and local anthropogenic influence (Gouello et al. 2021). The present results reinforce this principle: the industrial, agricultural, desert and coastal environments each harboured unique microbial assemblages functioning as natural biogeographic barcodes. Importantly, even sites that are geographically proximate, all situated within the Dubai metropolitan region exhibited discernible microbiome differences, underscoring a fine spatial resolution of microbial biogeography in this landscape. Prior research has demonstrated that soil samples from the same location resemble each other more closely than samples of comparable soil type from different locations (Gouello et al. 2021). We observed the same pattern: the site imprint consistently dominated over depth‐related variation in the ordination. This uniqueness of soil microbiomes can be harnessed in forensic investigations, enabling investigators to associate a trace soil sample with a particular environment or exclude it from others (Gouello et al. 2021). The depth‐related anomalies documented in this study, particularly the Desert deep Pseudomonadota reversal and the Human Activities Bacillota enrichment add a further layer of complexity: they suggest that the depth from which a soil sample was recovered may itself carry forensically informative signatures, provided that local reference databases capturing these depth‐specific patterns are available. The stark gradients and minimal vertical mixing characteristic of hyper‐arid environments may actually strengthen the forensic utility of microbial profiling in this region, as they promote and maintain compositional contrasts both horizontally across sites and vertically within profiles over ecologically relevant timescales.

The findings presented here offer a compelling proof of concept for the use of soil microbial profiles as environmental signatures in hyper‐arid settings. Broader validation across larger spatial scales, seasonal timepoints and greater sample sizes will be necessary before this approach can be reliably applied in operational contexts. Nevertheless, the consistency and distinctiveness of the community profiles documented across Dubai's contrasting land‐use types provide a strong foundation, and we see genuine potential in soil microbiome analysis as a complementary tool for forensic geolocation and environmental characterisation.

## 5 Conclusion

This study demonstrates that soil microbial communities in Dubai's hyper‐arid environment are shaped by two principal axes of variation: land‐use type and soil depth. Actinomycetota and Pseudomonadota co‐dominated across all sites, yet their relative proportions varied in ecologically meaningful ways with drought‐tolerant Actinomycetota prevailing in undisturbed desert surface and mid‐depth horizons, and copiotrophic Pseudomonadota enriched wherever moisture or organic inputs were elevated. The Marine and Desert sites exhibited the most distinctive microbiomes, driven by extreme salinity and aridity, respectively, while anthropogenically influenced sites showed predictable shifts towards nutrient‐responsive taxa. Notably, the Desert deep horizon (50–100 cm) and the Human Activities site revealed anomalous depth‐associated patterns: a Pseudomonadota reversal and a pronounced Bacillota enrichment, respectively, underscoring that vertical compositional gradients in hyper‐arid soils are not always linear and merit closer investigation. From a forensic standpoint, the site specificity of microbial fingerprints, reproducible even across geographically proximate locations within Dubai, supports the potential of soil microbiome profiling as a complementary geolocation tool. Further work involving larger reference datasets, temporal replication and functional gene characterisation will be needed to fully realise this potential, but the present findings establish a meaningful empirical foundation for both ecological understanding and applied forensic science in arid environments.

## Author Contributions

**Abdulla Albastaki:** methodology, writing – original draft, visualization, writing – review and editing. **Mohammed Naji:** writing – original draft, methodology, visualization, writing – review and editing. **Judith Smith:** writing – review and editing, supervision. **Mohamed Moussa:** writing – review and editing.

## Conflicts of Interest

The authors declare no conflicts of interest.

## Data Availability

The sequencing data supporting the findings of this study are not publicly deposited at this time but are available from the corresponding author upon reasonable request.
